# 

**Published:** 1991-06

**Authors:** 


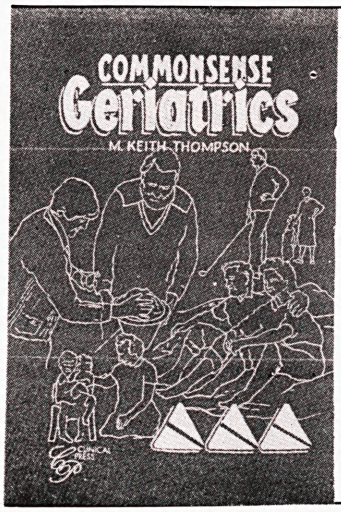

### Commonsense GERIATRICS

**Keith Thompson, General Practitioner, London**
**Examiner for the Diploma in Geriatric Medicine**

150 pages. Papercover. Fully illustrated. ISBN 1-85457-007-2. £12.50

*Clinical Press*

CP

### REGAINING BLADDER CONTROL

*Second edition*

*Clinical Press*

Eileen Montgomery, MCSP.,

For those suffering from incontinence on exertion or following pelvic surgery.

This important patient handbook contains a planned series of exercises aimed at restoring control of the bladder. The anatomical causes of incontinence are clearly explained and illustrated. The book also deals with correct breathing and a balanced diet which will not lead to constipation or to being overweight.

Surgeons, doctors and physiotherapists will be able to recommend this booklet to their patients.

28 pages. Soft cover. 11 line drawings. ISBN: 1 85457 003 X

Price inclusive postage and packing £2.50 per copy or £20.00 per pack of 10 copies.

Please order from your usual bookseller or direct from Clinical Press Ltd., c/o Gazelle Book Services, Falcon House, Queen Square, Lancaster, LA1 1RN, UK.

### THE CHESTERFIELD NUFFIELD HOSPITAL

### Your own private hospital

*Benefit from the experience...!*

For further details contact The Chesterfield Nuffield Hospital

3 Clifton Hill, Clifton, Bristol BS8 1BP. Telephone (0272) 730391

Nuffield Hospitals

### WEST OF ENGLAND MEDICAL JOURNAL

**JUNE 1991** **Volume 106 (ii)**

Copyright © West of England Medical Journal Ltd 1991.

**Editorial Committee**
Chairman of Editorial Board, Professor John Farndon
Editor— Mr M. G. Wilson
Assistant Editors—Dr Paul Goddard, Dr Alison Round
Secretary—Mr Clive Ward
Members—Dr John Burton, Dr Kenneth Southgate

**Editorial Office**—*The Red House, 40 Coombe Lane, W.O.T., Bristol BS9 2BJ*
*Tel 0272 682320*

**Correspondents**
Dr Andrew Adam (Taunton)
Mr Martin Crosfill (Truro)
Dr Jack Davies (Bristol)
Professor Denis Pereira Gray (Exeter)
Dr Jose Jancar (Bristol)
Dr Michael Oliver (Barnstaple)
Dr Michael Whitfield (Bristol)

**Associated Societies**
Bristol Medico-Chirurgical Society
Clinical Society of Bath
Severn Faculty of the Royal College of General Practitioners
South West of England and Wales Society of Dermatology
South West Obstetricians' and Gynaecologists' Club
South West Orthopaedic Club
South West Paediatric Club
South West Radiologists' Association
South West Urologists' Club
South West Vascular Surgeons
Surgical Club of South West England
Tamar Faculty of the Royal College of General Practitioners
Wessex Association of Clinical Pathologists
West Country Chest Physicians' Club
West Country Physicians' Club

Members of the above organisations receive the journal as part of their membership subscription.
Annual Subscription Rate £25.00 including surface postage.

**Published by**
Clinical Press,
*15 Bucklands Drive,*
*Nailsea,*
*Bristol BS19 2PH*

**Typesetting by**
*SuttonSet, Printing House, Barton Road, Torquay.*

**Printed by**
*The Devonshire Press Ltd, Printing House, Barton Road, Torquay*

**THE WEST OF ENGLAND MEDICAL JOURNAL acknowledges with gratitude the help it receives from NUFFIELD HOSPITALS**

**Contents**

1883–1991 Vols 1–104

**NOTICE TO CONTRIBUTORS**
Contributions are invited especially from West of England authors on a variety of subjects, e.g. Original articles relating to the practice of medicine, reports on the contemporary medical scene, obituaries, historical accounts, recollections of colleagues and events worthy to be remembered and liable to be forgotten. An article of 2,000 words with 2 illustrations will occupy 2 pages and this is a suggested, though by no means an obligatory length. Articles are preferred in the form proposed by the International Committee of Medical Editors (Vancouver System) — pamphlet obtainable from Editor of BMJ, BMA House, Tavistock Square, London, WC1H 9JR. Please send two copies retaining a further copy for your reference. **Articles published in this Journal are listed in Index Medicus.**

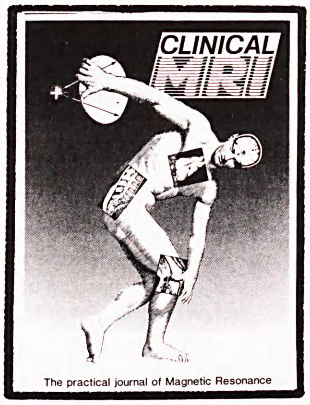

Two issues of ***Clinical MRI,***
***The Practical Journal of Magnetic Resonance***
**have now been published. Don't miss the next issue of this useful journal. Subscribe now !!!**

***UK PRICE: ONLY £ 30***

***PER ANNUM*** *from*

CLINICAL PRESS

*************************************

### CLINICAL FILM VIEWING SERIES:

### THE RESPIRATORY SYSTEM

**BY PAUL GODDARD MD,FRCR**

**This book will assist candidates to prepare for the final examination of the FRCR. The book consists of eighty profusely illustrated cases and many lists and flowcharts describing the modern practice of chest radiology. The text is readable and concise.**

***This is an invaluable learning book !*** *ISBN : 1 85457 014 5* *£ 15.00*

### THE CARDIOVASCULAR SYSTEM

**by GEORGE HARTNELL FRCR**

**"A well-organized presentation of case material which will help residents in radiology and other specialities to assess their knowledge of radiological diagnosis of cardiovascular disease"**
**A. de Roos MD, European Journal of Radiology**

*ISBN: 1 85457 012 9* *£ 15.00*

***COMING SOON : ULTRASOUND by KABALA and SLACK***

*ISBN: 1 85457 013 7* *£ 15.00*

*****************************************************************************

***For students of radiology and radiography***

### LECTURE NOTES ON

### THE PHYSICS OF RADIOLOGY

**by SUSAN ARMSTRONG FRCR**

The principles of radiological physics in a concise and comprehensive format

*ISBN: 1 85457 0102* *£ 15.00*

**Please order books from your usual bookseller or direct from Clinical Press Ltd.**
**c/o Gazelle Book Services Ltd ,Falcon House, Queen Square,Lancaster,LA1 1RN,UK**
***Clinical MRI*** **is available from Redland Green Farm, Redland, Bristol BS6 7HF,UK**

### ORDER YOUR COPY OF THE MEDICAL ANNUAL

### While stocks last!

THE MEDICAL ANNUAL

1991

The Yearbook of General Practice
Edited by John Fry
and Thomas Bouchier-Hayes

**Contents:**

Editorial - The Year in Retrospect GENERAL EVENTS
Medicopolitical Matters. Medicolegal Matters. HEALTH AND DISEASES
AIDS - The Impact on General Practice. Silent Myocardial Ischaemia
Thrombolytic Therapy in Emergency Treatment. Dyspepsia in the Community. Asthma. Diabetes Therapy, New Insulins, Non-insulin Drugs. Laser Hysterectomy. Recent Progress and Advances in Dermatology. Recent Progress and Advances in Viral Hepatitis.
Travel Medicine. Depression and Anxiety in General Practice. Sleep Disorders. THERAPEUTICS. he British Pharmaceutical Industry. Recent Advances in Drugs for Cardiovascular Disease.
Drugs in Peptic Disease. Drugs for Asthma. Recent Advances in Drugs for Dermatology. Hormone Replacement Therapy. PREVENTION AND HEALTH PROMOTION. Preventive Medicine and Health Promotion.
The Practice Nurse Past, Present and Future. Geriatrics. Minor Surgery in General Practice. NEW TECHNOLOGY. Magnetic Resonance Imaging. The Lithotripter. MISCELLANY. Facts - 1990: Personal Musings on General Practice. Great Medical Institutions - The Royal Society of Medicine. Index.

*250 pages approximately. 30 mm x 150 mm. Casebound.*
*ISSN: 0076 5899 ISBN: 1 85457 021 8*

### Rush me :-

Please order from your usual bookseller or direct from Clinical Press Ltd., Gazelle Book Services, Falcon House, Queen Square, Lancaster, LA1 1RN,

**MEDICAL ANNUAL 1991**

**The Year Book of General Practice**

£25.00

**Prepaid orders or credit-card orders will be supplied post free**

I enclose a cheque for ............. (Payable to Gazelle Book Services)

OR

I authorise you to charge my Access/Barclaycard/American Express credit account

Card: ....................................... Number: ....................................

Expiry Date: ............................ Signature: ..................................

OR

Please invoice me at list price plus £1.50 per copy postage and packing

Name: (Please Print) ..................................................................

Address: (Please Print) ..................................................................

